# A Rare Co-Occurrence of Triple Mutations in *JAK2, CALR*, and *MPL* in the Same Patient with Myelofibrosis

**DOI:** 10.1155/2022/4579122

**Published:** 2022-02-21

**Authors:** Sherine J. Thomas, D. P. Dash

**Affiliations:** ^1^Hematology and Oncology, Northside Hospital Cancer Institute, Georgia Cancer Specialists, Atlanta, GA, USA; ^2^Molecular Oncology & Genetics (MOGL), Versiti Wisconsin (Formerly Known as Blood Center Wisconsin), Milwaukee, WI, USA

## Abstract

*Background*. The diagnosis and prognostication of myeloproliferative neoplasm rely on the presence of driver mutations in JAK2, calreticulin (CALR), and MPL mutations. In the past, the presence of these mutations was thought to be mutually exclusive. Since then, there have been multiple reports of the presence of dual mutations. The presence of all three driver mutations in the same patient with myelofibrosis has not been previously described. Case. A 73-year-old female underwent a hematological workup in our facility after a routine hemogram performed prior to complex ophthalmological surgery revealed severe thrombocytosis. A comprehensive workup including an NGS panel for MPN driver mutations demonstrated that she had a calreticulin type-1 mutation, a JAK2 exon 14 (JAK2L611S) mutation, and an abnormal hotspot variant for MPL with VAF1%. A bone marrow biopsy confirmed a myeloproliferative neoplasm with grade 2 reticulin fibrosis suggesting primary myelofibrosis. Molecular profiling of bone marrow confirmed the previously noted mutations and an MPLW515R mutation. The patient was started on treatment with hydroxyurea and aspirin with improvement in platelet count and resolution of anemia. Discussion. The clinical significance of the presence of multiple driver mutations in the same patient is not well understood at this time. There have been 11 publications between 2014 and 2020 that have described dual mutations of JAK2V617F, MPL, and CALR mutations. The JAK2 exon 14 mutation noted, in this case, is JAK2L611S which has not previously been reported in MPN and only reported in 5 cases in the COSMIC database. The JAK2 exon 14 mutation identified in this case is not an established driver mutation for myeloproliferative neoplasm, and its clinical implication remains unknown. Conclusions. The above case in addition to recent case reports and case series supports the use of broader NGS sequencing panels for diagnosis and prognostication of MPN. These mutations should not be considered mutually exclusive. The clinical behavior and prognosis of the subgroup with multiple mutations need to be studied in larger series.

## 1. Introduction

The molecular basis of myeloproliferative neoplasms (MPNs) came into the limelight in 2005 with the identification of *JAK2* mutations in almost all cases of polycythemia vera and most essential thrombocythemia (ET) [1–4]. In 2006, somatic mutations in myeloproliferative leukemia virus oncogene (*MPL*) provided additional mechanistic understanding of the JAK-STAT pathway activation in MPN [5]. In 2013, somatic mutations in calreticulin (*CALR*) were reported as a driver mutation of essential thrombocythemia [6].

In clinical practice, these three driver mutations serve as diagnostic and prognostic markers during workup of myeloproliferative neoplasms. JAK2 mutations are present in most cases of polycythemia vera. Somatic mutation of codon 515 in exon 10 of the “myeloproliferative leukemia virus oncogene” (*MPL*) is found in an estimated 3-4% of patients with essential thrombocythemia (ET) and 7% of patients with primary myelofibrosis (PMF). Somatic mutations in calreticulin (CALR) are present in 25–30% of patients with essential thrombocytosis (ET) and primary myelofibrosis (PMF). CALR mutations have rarely been described in polycythemia vera. Importantly, mutations in *CALR* have previously been reported to be mutually exclusive with *JAK2* or *MPL* mutations [6–8].

More recently, several case reports and case series have reported the coexistence of two driver mutations in Philadelphia chromosome-negative MPN, but these are known to be rare events. The presence of triple mutations in *JAK2*, *CALR*, and *MPL* in the same patient with MPN has not been previously reported, and therefore, here, we intend to present this unique case report.

## 2. Case

A 73-year-old African American female with a medical history of glaucoma and cataracts presented for hematology evaluation after routine hemogram performed before eye surgery demonstrated thrombocytosis and anemia. Her hemogram demonstrated hemoglobin of 9.9 g/dL and hematocrit of 31.1%. WBC count was normal at 9300/mcL, absolute neutrophil count was 5952 mcg/dL, basophils were 3% with absolute basophil count 279/mcL, monocytes were 14%, and absolute monocyte count was 1302/microliter. Platelet count was elevated to 750,000/mcL. She underwent workup for iron deficiency which showed a normal iron panel. Evaluation for myeloproliferative disorder (MPN) driver mutations was also performed.

The patient's peripheral blood sample was obtained for targeted gene sequencing. DNA extracted from peripheral blood was amplified via PCR with primers specific for gene targets—*ABL1, ASXL1, BCOR, BRAF, CLAR, CDKN2A, CBL, CEBPA, CSF3R, DNMT3A, ETV6, EZH2, FLT3, GATA2, HRAS, IDH1, IDH2, FBXW7, IKZF1, JAK2, KIT, KRAS, MPL, MYD88, NF1, NPM1, NRAS, PHF6, PTEN, PTPN11, PRPF8, RB1, RUNX1, SETBP1, SF3B1, SH2B3, SRSF2, STAG2, TET2, TP53, UAF1, WT1,* and *ZRSR2.* Specific genomic regions were targeted using a customized AmpliSeq Myeloid Next-Generation Sequencing (NGS) panel (Illumina), and sequencing by synthesis chemistry with paired-end 201 base pair read was performed. Bioinformatics sequence analysis pipeline was customized to limit analysis for only MPN gene targets.

Gene targets analyzed included all exons for *CALR* and selected exons with a high prevalence for the presence of pathogenic variants *JAK2* (exons 12–15) and *MPL* (exons 3, 4, 10, and 12).

The following abnormal gene alterations were identified:

1. *CALR* p.(Leu367ThrfsTer46) with a variant allele frequency of 46.5% (c.1099_1150delCTTAAGGAGGAGGAAGACAAGAAACGCAAAGAGGAGGAGGAGGCAGAGG) (52 bp deletion, type 1).
2. She was also found to have *JAK2* p.(Leu611Ser) with variant allele frequency 23.2% (c.1832T > *C*).
3. In addition to the above, abnormal testing for hotspot variant *MPL* p. Trp515Arg was detected with VAF 1%, which is below the level of detection for that test (1.75%): variant depth 56 and total depth 5554. Confirmatory testing with higher sensitive PCR-based locked nucleic acid (LNA) demonstrated the same *MPL* mutation (p. Trp515Arg (W515R)) (tier I variant: NM_005373.2). The confirmatory testing for *MPL* exon 10 mutation analysis is validated based on locked nucleic acid (LNA) methods for the *MPL* W515R codon which offers a higher sensitivity than traditional Sanger sequencing assays. Based on the LNA methods of detection, the higher sensitive MPL assay can detect mutation at the *MPL* W515R codon with low allele burden as low as 2% or below.

Given the unusual genetic mutation profile, a bone marrow biopsy was undertaken to quantify bone marrow fibrosis.

Flow cytometry on the bone marrow aspirate specimen failed to identify any unique cell populations of myeloid or lymphoid origin. A small population of cells identified by surface markers, CD13, CD22, low-density CD20 5, moderate CD33, bright CD38, moderate CD45, bright CD123, and negative for CD19, and by side scatter properties as basophils were noted to comprise 2% of the total nucleated sample. Monocytes were 6% of the nucleated sample, granulocytes 59%, and lymphocytes 25%.

**Figure 1:**
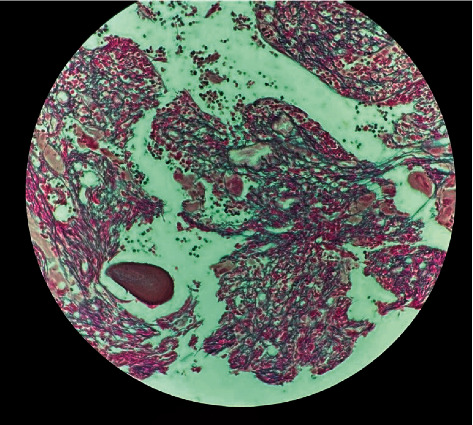
Reticulin-stained bone marrow core biopsy showing increased reticulin fibrosis. WHO^*∗*^ classification MF2 (^*∗*^World Health Organization).

**Figure 2:**
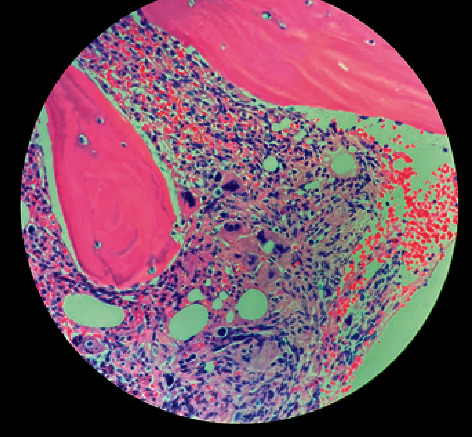
H&E-stained section of bone marrow core biopsy showing atypical megakaryocytic hyperplasia.

Morphologically, the core biopsy demonstrated a hypercellular marrow (95% cellularity) with trilineage hematopoiesis, myeloid-to-erythroid ratio >5 : 1, with atypical megakaryocytic hyperplasia, moderately increased fibrosis, but no increase in blasts (Figures 1 and 2). Karyotype was noted to be normal-46, XX [20].

Based on the above workup, the patient was diagnosed with chronic myeloproliferative neoplasm-primary myelofibrosis.

**Figure 3:**
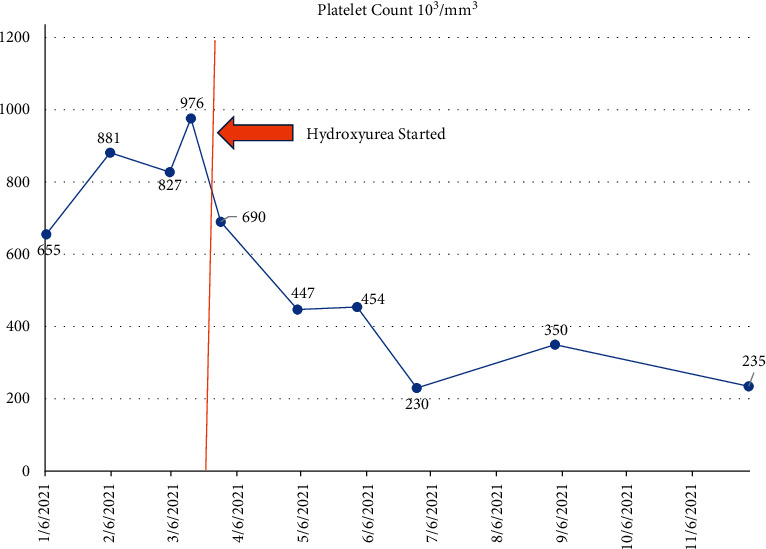
Graph of the platelet count at presentation and response to hydroxyurea.

**Figure 4:**
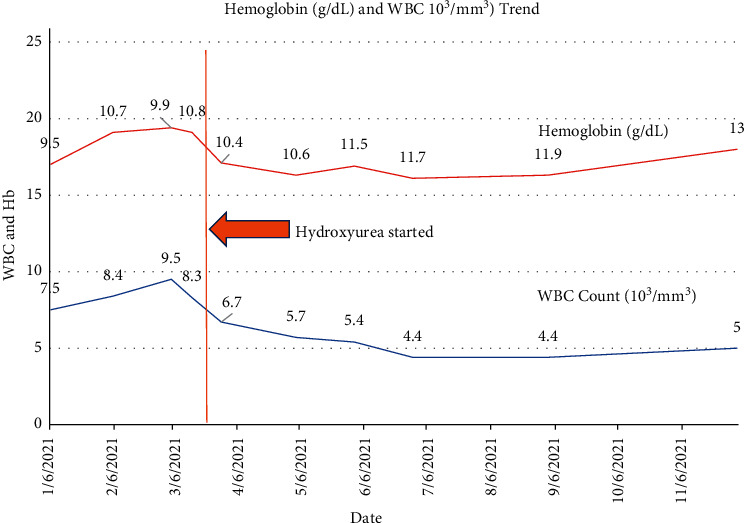
Graph of hemoglobin and white blood cell count trend and response to hydroxyurea.

The patient was started on treatment with hydroxyurea for cytoreduction and on aspirin for thromboprophylaxis, with improvement in the platelet count (Figure 3). She had improvement and normalization of hemoglobin (Figure 4).

Peak platelet count was 976,000/mcL at the time of initiation of cytoreductive therapy with hydroxyurea. Within 4 months of starting cytoreduction with hydroxyurea, the patient was noted to have normalization of the platelet count to 220,000/mcL. WBC count and neutrophils remained stable, and anemia resolved. The patient remained stable and continued to tolerate hydroxyurea therapy. She has remained on aspirin for thromboprophylaxis.

## 3. Discussion

The presence of driver mutations has diagnostic, prognostic, and therapeutic implications in myeloproliferative disorders. To our knowledge, this is the first case of a patient with ET identified to have triple mutations *JAK2* L611S*, CALR* type 1, and tier 1, *MPL* W515R, co-occurring in the same patient.

As mentioned above, the presence of multiple driver mutations in patients with MPN is well described, but they are rare events. For example, in a study evaluating the prevalence of MPN driver mutations in 928 Chinese patients, the *JAK2* V617F and *CALR* exon 9 dual mutation was identified in six (0.6%) patients, and the coexistence of *JAK2* V617F and *MPL* exon 10 was even more uncommon, occurring in two (0.2%) patients [9].

*JAK2* V617F mutation was the first and the most well-characterized driver mutation in myeloproliferative neoplasm. The patient described in this report had a less common *JAK2* p.L611S mutation. We note that *JAK2* p.L611S mutation is not a recognized driver mutation in MPN. However, given the clinical relevance of *JAK2* mutations in MPN, we feel that this patient's less common *JAK2* mutation profile should be described.

### 3.1. *JAK2* p.L611S Mutation

Janus kinase 2 (*JAK2*) helps in modulating response to extracellular cytokine signalling. While *JAK2* is a nonreceptor TKI, it needs a cognate receptor for response. Activated *JAK2* signalling is necessary for normal hematopoiesis including erythropoiesis and thrombopoiesis. Once activated, *JAK2* triggers recruitment and phosphorylation of downstream molecules such as STAT3/5 and MAP kinase which in turn allows the translocation of signalling molecules to the nucleus and thereby activates transcription of functional proteins.

*JAK2* p.L611S is a missense mutation in exon 14 of the *JAK2* gene in chromosome 9. It results in the replacement of amino acid leucine to serine at codon 611 (p.L611S). This variant represents a hotspot mutation within the JAK2-JH1 interface including one in the JH2 domain (L611S) and is supposed to destabilize JH2-JH1 interaction and enhance *JAK2* signalling.

*JAK2* p.L611S has been reported in the COSMIC database (COSV67633764, legacy identifier: COSM21361) for a total of 4 cases including PV (with comutation in *JAK2* V617F), AML in conjunction with Down syndrome myeloid disorder (DS-AMKL) (comutated with *GATA* and *NIPBL* and 7qdel, B-ALL, and squamous cell carcinoma (COSM21361, February 2021). *JAK2* p.L611S has been shown to be associated with tumorigenesis in animal models [10]. Although *JAK2* p.L611S mutation has not been reported with myelofibrosis or essential thrombocytosis, it has been reported in a child with congenital thrombocytosis.

### 3.2. Coexistence of Multiple Driver Mutations

Most cases of myeloproliferative neoplasm are known to harbour driver mutations *JAK2*, *MPL*, or *CALR* in a mutually exclusive manner [11]. However, there have been cases of rare patients with 2 coexisting mutations.

Co-occurrence of *CALR* and *MPL* has been reported in one patient from India [12]. Similarly, a case report from South Korea of 123 MPN patients reported the presence of *JAK2* and *CALR* mutations in 7 (4%) patients [13]. Another case from Korea in 2021 reported a case of comutation of *JAK2* V617F and MPL mutation [14].

A case series of 11 MPN patients published in 2018 with multiple driver mutations (including *BCR-ABL*) had 8 patients positive for *BCR-ABL* and *JAK2* V617F mutations, 1 patient positive for *BCR-ABL* and *CALR* type 2 mutations, and 2 patients (referenced in Table 1) negative for Philadelphia chromosome, and one each among them had *JAK2* V617F *+* *MPL*, *JAK2* V617F, and *CALR* mutations [22].

The clinical behaviour of patients with these rare comutations is not well understood because of the rarity of these events. The limited case reports regarding these comutations have provided some insights.

A previous case series has suggested that the presence of *JAK2* and *CALR* did not appear to affect the prognosis or clinical features of ET, thereby suggesting that the disease phenotype is dictated primarily by the *JAK2* mutation. They also noted that ET patients harboring mutations in the *JAK2* gene had inferior progression-free survival (PFS) regardless of the presence of a mutation in *CALR*. They also suggested that response to therapy was superior in patients bearing *JAK2* mutations as compared to patients with *CALR* mutations [16].

Authors from a separate series suggested that the double mutated phenotype has a different clinical course compared to those with a single driver mutation [15].

Another larger case series of 40 double mutated patients with essential thrombocythemia showed that these patients with multiple mutations have a male predominance, the advanced age of onset, lower hemoglobin, and higher platelet count compared to the patients with a single mutation. However, the incidence of thrombosis was not different compared to patients with a single driver mutation [23].

The *JAK2* p.L611S mutation identified in this case has not been recognized as a driver mutation of myeloproliferative neoplasm. However, given the seminal role of *JAK2* mutations in these diseases, this patient's mutation profile is of interest.

## 4. Conclusion

To the best of our knowledge, this represents the first reported case of the coexistence of *JAK2* p.L611S*, CALR* type 1, and tier 1 *MPL* mutation in the same patient with essential thrombocythemia. It is also the first reported case of *JAK2* p.L611S in an adult with myelofibrosis. This case report highlights the increased recognition of cases with multiple mutations in *JAK2*, *MPL*, and *CALR*. Longer-term follow-up of patients with these comutations will lead to a greater understanding of their clinical behaviour and prognosis.

## Figures and Tables

**Table 1:** A summary of case reports and case series with multiple driver mutations present in patients with ET and PMF.

No.	Author	*N*	MPN	JAK2 exon 14	JAK2 exon 12	MPL	CALR	Year
1	McGaffin et al. [15]	1	—	V617F	—	—	Type 6 (48 bp del)	2014
2	Kang et al. [16]	7	ET	V617F	—	—	All types (1, 2, and3)	2016
3	Rashid et al. [17]	1	ET	V617F	—	—	Type 1 (52 bp del)	2016
4	Cleyrat et al. [18]	1	ET	—	—	p.W515R	Type 1 (52 bp del)	2017
5	Jeromin et al. [19]	12	—	V617F	—	Type not reported	—	2017
		6	—	V617F	—	—	Type unreported	
		1	—	—	—	Type not reported	Type unreported	
6	Usseglio et al. [20]	3	ET	V617F	—	W515L	—	2017
		1	ET	V617F	—	W515R	—	
		4	ET	V617F	—	—	Type 1 (52 bp del)	
7	Boddu et al. [21]	1	ET	V617F (VAV <1%)	—	—	Type 1 (52 bp del)	
8	De Roeck et al. [22]	1	ET	V617F	—	p.W515R	—	2018
		1	PMF	V617F	—	—	Type 1 (52 bp del)	
9	Mansier et al. [23]	5	—	V617F (VAF <5%)	—	—	Type unreported	2018
		32	—	V617F	—	—	Type unreported	
		11	—	V617F	—	Type not reported	—	
		2	—	—	—	Type not reported	Type unreported	
		1	—	V617F	Type not reported	—	2 CALR mutations	
10	Ramanan et al. [12]	1	—	—	—	p.W515R (VAF 9.8%)	Type 1 52 bp deletion (VAF 13%)	2019
11	Zhou et al. [13]	1	PMF	—	—	p.X636W	CALR-p.364fs	2020

Table 1 is modified from Table 1 of Ramanan et al. [12].

## Data Availability

The data used to support the findings of this study, including high-resolution microscopic images and molecular test results, are included within the article.
